# A Pharmacoepidemiological Indicator for Detecting Potential Regional Overuse of Hypnotics in Japan: A Cross-Sectional Study

**DOI:** 10.7759/cureus.96960

**Published:** 2025-11-16

**Authors:** Ryosuke Shinkai, Takashi Tomita

**Affiliations:** 1 School of Pharmacy, International University of Health and Welfare (IUHW) - Narita Campus, Narita, JPN; 2 Pharmacy, International University of Health and Welfare (IUHW) - Narita Hospital, Narita, JPN; 3 Pharmacy, International University of Health and Welfare (IUHW) - Mita Hospital, Minato, JPN

**Keywords:** cluster analysis, hypnotics, overprescribing, pharmacoepidemiology, prescriptions, sunlight exposure

## Abstract

Objectives

The main objective of this study is to examine how environmental and demographic factors shape regional prescription patterns of circadian-related hypnotics, and to develop a pharmacoepidemiological indicator for detecting potential regional overuse.

Methods

A nationwide ecological cross-sectional study was conducted using 2022 data from all 47 prefectures of Japan. Population-adjusted prescription volumes of ramelteon, suvorexant, and lemborexant were obtained from the National Database of Health Insurance Claims. Environmental (annual sunlight duration and ultraviolet index) and demographic (aging rate and outpatient clinic density) variables were extracted from governmental statistics. Pearson correlation and multivariable linear regression analyses were conducted, and residuals were geographically visualized to identify regional deviations from model expectations.

Results

Ramelteon and suvorexant prescriptions were significantly associated with shorter sunlight duration and higher aging rates (r = -0.64 and -0.61, both p < 0.001). In multivariable analysis, sunlight duration and aging rate independently predicted prescription volumes (adjusted R² = 0.46), with improved performance (adjusted R² = 0.51) after exclusion of high-influence outliers. Residual analysis revealed that unexplained positive deviations were concentrated in urban prefectures, suggesting locally elevated prescribing tendencies.

Conclusions

Regional prescription volumes of circadian-related hypnotics can, to some extent, be anticipated from environmental determinants such as sunlight duration. The proposed indicator-based pharmacoepidemiological framework may help identify regions at risk of potential overuse and guide climate-sensitive strategies for optimizing hypnotic use in older adults.

## Introduction

Sleep disorders are a major concern in aging societies, as sleep quality declines with age and affects physical and cognitive health. Insomnia is common among older adults [1,2], and safer pharmacologic alternatives to benzodiazepines are increasingly needed [3,4]. In Japan, the burden of insomnia has social and economic implications, and national health policies have increasingly emphasized safer prescribing for older adults through initiatives promoting the use of non-benzodiazepine hypnotics. These efforts highlight the importance of understanding regional prescribing behaviors in relation to environmental and demographic factors. Circadian rhythm-modulating agents, including the melatonin receptor agonist ramelteon and the orexin receptor antagonists suvorexant and lemborexant, have recently gained clinical prominence, particularly in geriatric care settings [5,6]. These drugs promote physiological sleep and have more favorable safety profiles than traditional hypnotics.

Epidemiological studies have shown that reduced sunlight exposure is associated with delayed sleep phase, lower melatonin secretion, and increased prevalence of insomnia symptoms in community-dwelling populations, particularly during winter or at higher latitudes [7,8]. In Japan, prefectures with shorter daylight duration also report higher rates of depressive and sleep-related symptoms, suggesting that environmental light deprivation contributes to circadian misalignment and sleep demand. Under such conditions, pharmacological interventions such as melatonin receptor agonists or orexin antagonists are often selected over nonpharmacological options like light therapy in primary care settings, especially among older adults who have limited outdoor exposure or difficulty maintaining regular sleep-wake schedules.

Although these circadian-modulating hypnotics are generally safer than benzodiazepines, recent pharmacoepidemiological studies and post-marketing surveillance have reported their prolonged or inappropriate use, particularly among older adults. Such observations suggest that even safer alternatives can be overprescribed or used beyond guideline recommendations, highlighting the importance of monitoring potential regional overuse.

Because they act on endogenous circadian systems, their prescription may be influenced by environmental factors such as sunlight exposure and the day-night rhythm. For instance, ramelteon mimics light-regulated melatonin secretion, and orexin receptor antagonists also modulate sleep-wake transitions through circadian mechanisms. Although numerous laboratory studies have shown that light exposure strongly affects circadian regulation and sleep physiology [9-11], large-scale pharmacoepidemiological analyses linking environmental light and real-world prescribing behavior remain limited. Understanding this relationship is important not only for clinical decision-making but also for developing region-sensitive prescription surveillance systems that account for demographic and environmental contexts. Therefore, this study aimed to examine the environmental and demographic determinants of circadian rhythm-modulating hypnotic prescriptions across all 47 prefectures of Japan and to explore a pharmacoepidemiological indicator capable of detecting potential regional overuse. This indicator is exploratory in nature and derived from residual modeling, serving as a preliminary framework to identify potential regional overuse rather than a validated metric.

In this context, “potential regional overuse” refers to prefectural prescription volumes that substantially exceed levels expected from demographic (e.g., aging rate) and environmental (e.g., sunlight exposure) conditions, as estimated by our multivariable model. Thus, higher positive residuals represent areas with relatively greater use than predicted, serving as a preliminary signal of possible overprescription at the regional level.

## Materials and methods

Study design

This cross-sectional ecological study included regional data on environmental factors and prescription volumes of circadian rhythm-modulating hypnotics between January 1 and December 31, 2022, from all 47 prefectures of Japan [12].

Data sources and variables

Prescription data were extracted from the 2022 edition of the National Database of Health Insurance Claims and Specific Health Checkups of Japan Open Data (NDB Open Data), provided by the Ministry of Health, Labor, and Welfare [11]. The target drugs were ramelteon, suvorexant, and lemborexant, which are increasingly prescribed to older adults as non-benzodiazepine alternatives [13,14]. The environmental factors assessed included the prefecture-level annual sunlight duration, published by the Japan Meteorological Agency [15], and the average annual ultraviolet (UV) index data, provided jointly by the Ministry of the Environment and Japan Meteorological Agency [16]. Additional demographic and healthcare variables included the aging rate (percentage of the population aged ≥65 years) and the number of outpatient clinics per 100,000 population, obtained from the Statistics Bureau of Japan [17]. The study population included all age groups covered by Japan’s National Health Insurance system; however, the analysis particularly focused on prescription trends among older adults (aged ≥65 years), who represent the main target population for non-benzodiazepine hypnotics.

Outcome and standardization

The annual prescription volume for each hypnotic was standardized per 100,000 people at the prefecture level. Prefectural population data used for standardization were obtained from the 2022 Population Census by the Statistics Bureau of Japan, and descriptive statistics for all prefectures are provided in Appendix, Table 2. All variables were based on official statistics compiled for 2022.

Statistical analysis and geographic visualization

Univariate associations between the prescription volumes of ramelteon, suvorexant, and lemborexant and each explanatory variable - annual sunlight duration, UV index, aging rate, and outpatient clinic density - were assessed using Pearson’s correlation coefficients. Multivariate linear regression models were constructed, wherein annual sunlight duration, aging rate, and outpatient clinic density were considered explanatory variables, to estimate prescription volumes. The model fit was evaluated using the adjusted coefficient of determination (adjusted R²). Residuals (observed minus predicted values) were computed to identify deviations from model expectations and visualized as heatmaps to illustrate regional disparities. Standardized regression coefficients, along with their 95% confidence intervals and p-values, are provided in the Appendix, Tables 4-5. Heatmap values were categorized into 10 quantiles, with darker shades indicating stronger values. Positive residuals are rendered in warm colors, and negative residuals in cool colors [18]. Thus, prefectures shown in warmer (red-orange) colors represent higher-than-expected prescription volumes, whereas cooler (blue-green) colors indicate lower-than-expected values. This color-coding facilitates intuitive interpretation for readers unfamiliar with geospatial statistical maps. All statistical analyses were performed using R (version 4.3.0; The R Foundation for Statistical Computing, Vienna, Austria) and EZR (version 1.61) [19]. Spatial distribution maps of prescription volumes and residuals were created using QGIS (version 3.28) [20]. All prescription volumes were standardized per 100,000 population using official prefectural census data to allow inter-prefectural comparison. Multicollinearity was examined using the variance inflation factor (VIF), confirming that all VIF values were below 5. Outlier influence was assessed via Cook’s distance, with values exceeding 4/n flagged as influential and subjected to sensitivity analysis. Prefectures identified as having Cook’s distances exceeding the threshold (4/n) were excluded only in a sensitivity analysis to evaluate the potential influence of these outlying regions on regression estimates. These prefectures exhibited unique demographic and healthcare characteristics rather than data errors. The main model retained all 47 prefectures to ensure representativeness and comparability across Japan. Diagnostic plots were visually inspected to ensure linearity and homoscedasticity. These data-preprocessing and diagnostic procedures are summarized in the Appendix, Table 3.

Ethical considerations

This study used only publicly available, aggregated data containing no personally identifiable information. Therefore, in accordance with the national guidelines, formal ethical reviews and informed consent were not required.

## Results

Study sample and overview

Three circadian rhythm-modulating hypnotics - ramelteon, suvorexant, and lemborexant - were analyzed. Prescription composition ratios varied substantially across prefectures, although total volumes showed no major regional differences. Because sunlight duration was stable from 2018 to 2022, data from 2022 were used for analysis (Appendix, Figure 7).

Geographic variation and sunlight duration

**Figure 1:**
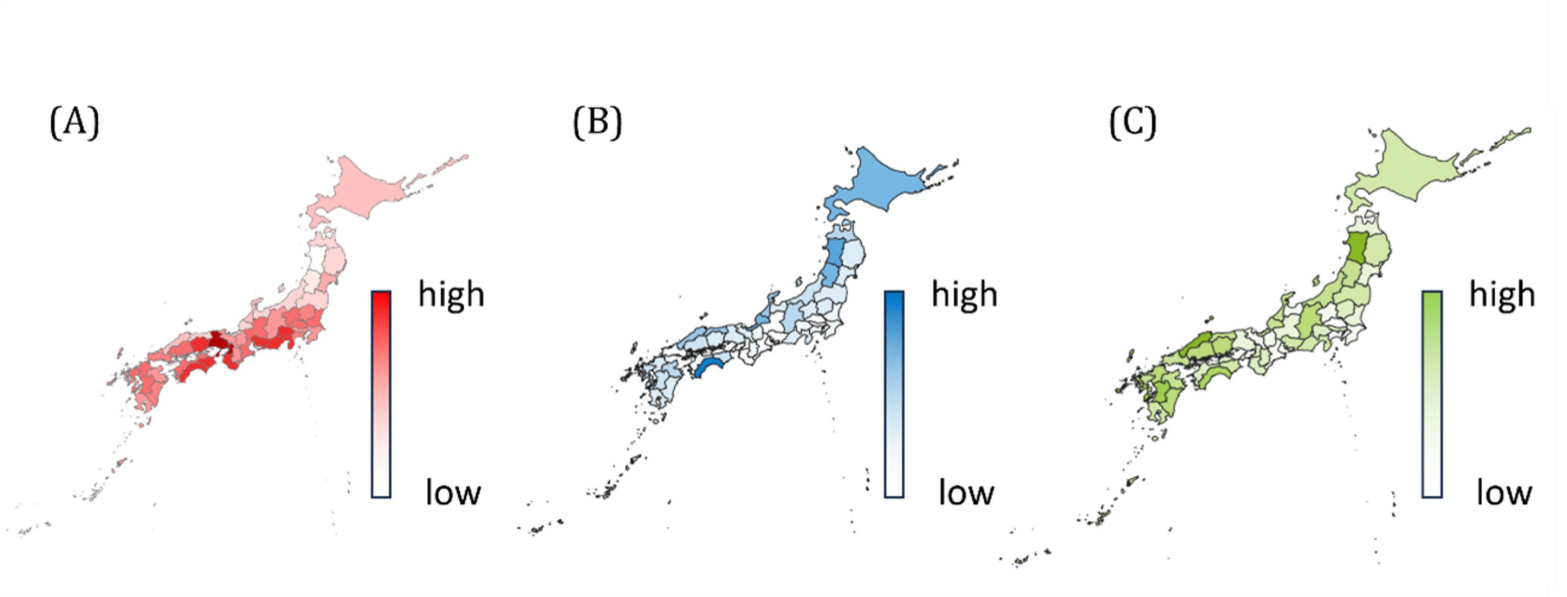
Geographic distribution of sunshine duration and circadian drug prescriptions in Japan (A) Heatmap of sunshine duration by prefecture. Data were obtained from the Japan Meteorological Agency using total annual hours recorded at representative meteorological sites (typically located in prefectural capitals). The color gradient reflects regional variations, with longer sunshine durations observed in western Japan and shorter durations in Hokuriku, Tohoku, and Hokkaido. (B) Heatmap of melatonin receptor agonist prescriptions per capita. This heatmap illustrates the population-adjusted prescription volume of the melatonin receptor agonist ramelteon by prefecture. Prescription data were retrieved from the National Database of Health Insurance Claims and Specific Health Checkups of Japan (NDB), and population estimates were obtained from the Statistics Bureau of Japan. Prescriptions per 100,000 individuals were calculated and visualized using a color gradient. An inverse pattern relative to sunshine duration was observed, with higher prescription densities in areas with shorter sunlight exposure. (C) Heatmap of orexin receptor antagonist prescriptions per capita. This heatmap shows the population-adjusted prescription volumes of the orexin receptor antagonists suvorexant and lemborexant by prefecture. Prescription volumes per 100,000 people were calculated using NDB and national census data. While partially mirroring the distribution of melatonin receptor agonists, prescription densities were relatively higher in certain urban areas, suggesting the influence of medical accessibility and regional demographic characteristics.

**Figure 2:**
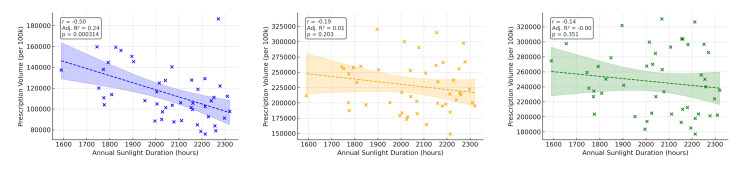
Correlation between sunshine duration and per capita prescription volume This scatter plot depicts the association between sunshine duration and the population-adjusted prescription volume (number of tablets per 100,000 people) of three circadian rhythm-regulating hypnotics: ramelteon, suvorexant, and lemborexant. Linear regression analyses were conducted for each drug, and the correlation coefficient (r), p-value, and adjusted R² are presented. A significantly negative correlation was observed for ramelteon, suggesting a higher prescription rate in areas with lower sunlight exposure.

**Figure 3:**
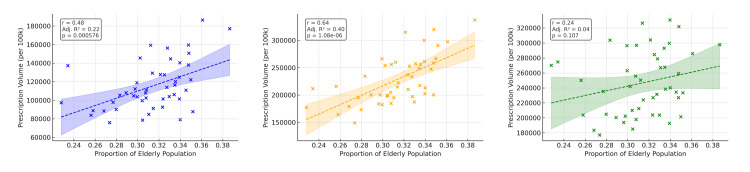
Correlation between aging rate and per capita prescription volume This scatter plot shows the relationship between the proportion of older individuals (≥65 years) and the per capita prescription volume for each drug. Data on the aging rate were obtained from the Basic Resident Register of the Statistics Bureau of Japan. Linear regression analysis revealed significant positive correlations for suvorexant and lemborexant, indicating their preferential use in older populations.

**Figure 4:**
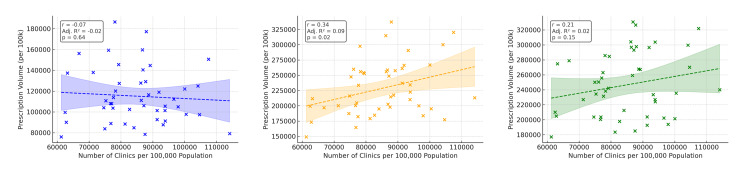
Correlation between the number of clinics and per capita prescription volume This scatter plot presents the association between the number of general outpatient clinics per prefecture and population-adjusted prescription volume for each drug. Clinical data were obtained from the Ministry of Health, Labour, and Welfare. A positive association was observed overall, with greater prescription volumes in prefectures with more clinics; however, drug-specific variability suggests that regional characteristics should be considered during interpretation. A significant negative association was observed between ramelteon use and duration of sunshine.

Prefectures with shorter annual sunlight duration tended to have higher prescription volumes of melatonin and orexin receptor modulators (Figure 1). Univariate regression using sunlight duration, aging rate, and outpatient clinic density (Figures 2-4) showed that ramelteon prescriptions were negatively associated with sunlight duration and positively associated with the aging rate. Suvorexant correlated positively with aging rate and clinic density, while lemborexant showed no significant association (Appendix, Table 4). The UV index, though correlated with sunlight duration (r = 0.52, p < 0.001), was not associated with total prescription volume.

Multivariable analysis of associated factors

**Figure 5:**
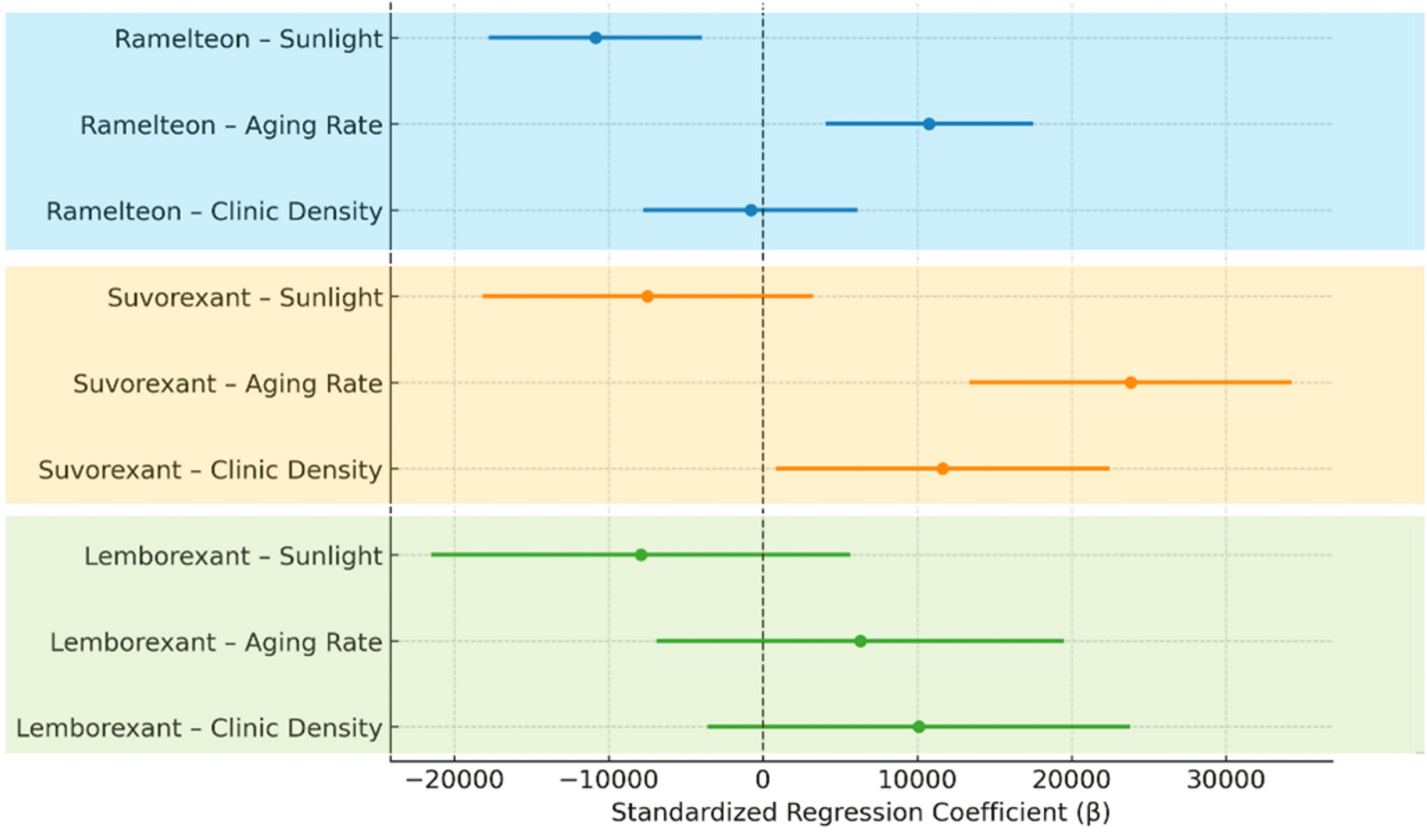
Multivariable regression analysis: forest plot of standardized coefficients This forest plot visualizes the associations between the population-adjusted prescription volume of circadian rhythm-regulating hypnotics and explanatory variables (aging rate, sunshine duration, and number of clinics), as estimated using multivariable linear regression. Each standardized regression coefficient (β) is presented with its 95% confidence interval. The results are color-coded according to the drug. Sunshine duration consistently showed a negative association with all drugs, whereas aging rate and clinic availability were positively associated with drug-specific variability.

Multivariate regression confirmed that sunlight duration and aging rate independently predicted prescription volumes of ramelteon and suvorexant, whereas outpatient clinic density contributed modestly. Lemborexant showed no significant associations, consistent with its higher use among younger adults. The adjusted R² values were 0.44 for suvorexant and 0.37 for ramelteon, indicating moderate explanatory power (Figure 5 and Table 1). These findings suggest that environmental and demographic factors, particularly sunlight duration and aging structure, partly account for regional prescribing behavior and may form the basis of an indicator of potential overuse.

**Table 1:** Results of multivariable regression analyses for ramelteon and suvorexant Regression coefficients (β), 95% confidence intervals, p-values, and adjusted R² values are shown for models including all prefectures (Figure 6A) and excluding influential prefectures (Figure 6B).

Figure 6A (All prefectures)				
Variable	Beta	95% CI	p-value	Adjusted R^2^
Sunlight duration	-95.0736	-172.6 to -17.5	0.01744	0.461185
Aging (older) rate	1068329	618993.5 to 1517663.7	1.98 × 10^-5^	0.461185
Clinics per 100,000	0.903437	-0.4 to 2.2	0.154	0.461185
Figure 6B (Excluding influential prefectures)				
Variable	Beta	95% CI	p-value	Adjusted R^2^
Sunlight duration	-77.2972	-156.2 to 1.6	0.054635	0.511079
Aging (older) rate	1006119	537942.5 to 1474296.5	9.58 × 10^-5^	0.511079
Clinics per 100,000	1.692624	0.6 to 2.8	0.004633	0.511079

Residual and outlier analysis

**Figure 6:**
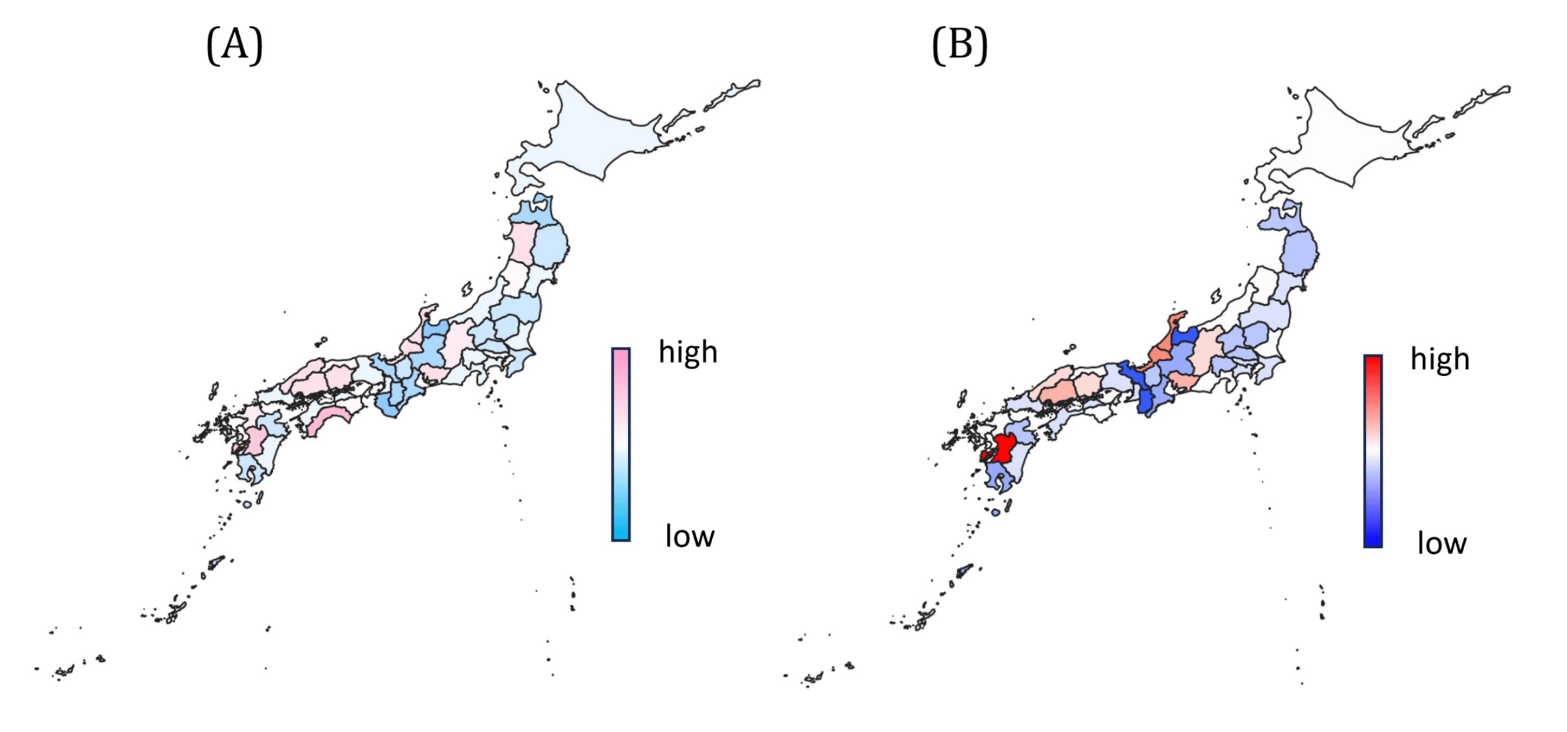
Residual heatmap from multivariable regression analyses of ramelteon and suvorexant (A) Residual heatmap of multivariable models for ramelteon and suvorexant. This heatmap displays the residuals (observed minus predicted values) from the multivariate regression models for ramelteon and suvorexant by prefecture. Residuals reflect the portion of variation not explained by the model, where positive values indicate potential over-prescription and negative values indicate under-prescription. This map offers insight into the regional model fit, with notably larger residuals in major metropolitan areas and northern Japan. (B) Residual heatmap after exclusion of influential prefectures. This heatmap shows the residual distribution following the exclusion of influential prefectures identified by a high Cook’s distance. The revised model yields a more uniform residual pattern, enhancing generalizability and minimizing region-specific influences.

**Figure 7:**
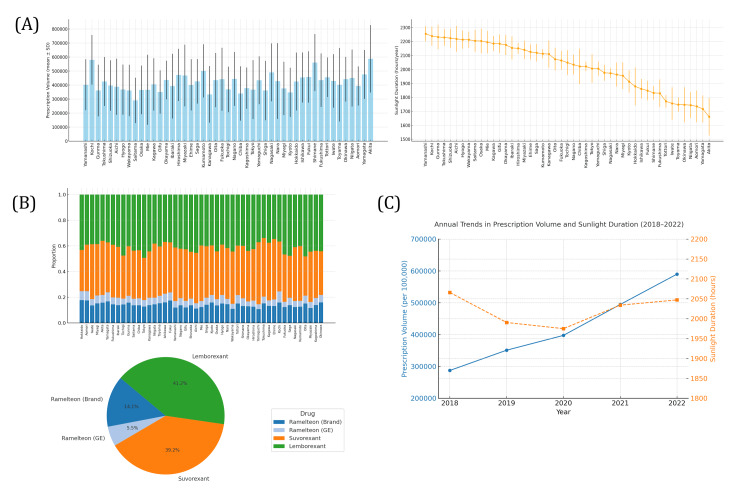
Regional distribution of circadian hypnotic prescriptions and medical infrastructure in Japan (2018-2022) (A) Prefecture-level total prescriptions (2018-2022): This bar graph illustrates the total annual number of prescriptions (number of tablets) for three circadian rhythm-regulating hypnotics - ramelteon, suvorexant, and lemborexant - across 47 Japanese prefectures between 2018 and 2022. Prescription data were extracted from the National Database of Health Insurance Claims and Specific Health Checkups of Japan (NDB), and the number of prescriptions per drug was aggregated by prefecture. The relative rankings among prefectures remained largely consistent over the five-year period, indicating stable regional variations in prescription volumes. (B) Prefecture-level number of general clinics (2018-2022): This bar graph presents the number of general outpatient clinics (regardless of specialty, including internal medicine and psychiatry) across Japan’s prefectures between 2018 and 2022. Data were obtained from the Survey of Medical Institutions by the Ministry of Health, Labor and Welfare, based on the number of facilities as of October 1 each year. The distribution of clinics remained relatively stable over time, with patterns suggesting associations with population density and urbanization. (C) Annual trends in prescription volume and sunshine duration: National averages of annual prescription volumes (number of tablets) for ramelteon, suvorexant, and lemborexant, along with the corresponding annual sunshine duration. Prescription data were derived from the National Database of Health Insurance Claims and Specific Health Checkups of Japan (NDB) and sunshine data from the Japan Meteorological Agency. Both indicators remained stable throughout the five-year period, supporting the selection of a single year for the cross-sectional analysis.

Because ramelteon and suvorexant displayed similar patterns, their combined prescription volumes were modeled together. Residuals (observed - predicted values) were visualized by prefecture (Figure 6A). The model yielded an adjusted R² of 0.46, outperforming individual drug models. After excluding four high-influence prefectures (Akita, Wakayama, Kochi, and Okinawa), identified by Cook’s distance, model fit improved (adjusted R² = 0.51) and coefficient stability increased (Figure 6B). These prefectures were not removed from the primary model but examined separately to assess robustness and regional heterogeneity. The resulting model represents a preliminary pharmacoepidemiological indicator capable of detecting deviations from expected prescribing behavior.

## Discussion

This nationwide ecological study showed that regional prescription patterns of circadian rhythm-modulating hypnotics are substantially shaped by environmental and demographic factors. Prefectures with shorter sunlight exposure and older populations exhibited higher prescription volumes of ramelteon and suvorexant, whereas lemborexant showed no such association, indicating that local environmental conditions and age structure measurably contribute to hypnotic use in Japan. Light is a primary zeitgeber for human circadian regulation, and reduced exposure plausibly intensifies sleep-wake disruption and demand for circadian‐targeting agents. At the physiological level, melatonin dynamics and circadian phase are sensitive to photic input, providing a mechanistic bridge between sunlight duration and prescribing behavior [21]. Beyond biology, sociobehavioral and structural contexts likely amplify regional variation. Urban prefectures, with dense healthcare infrastructure and a higher prevalence of shift work, may show elevated use independent of light exposure, while rural settings, with more outdoor activity, may show lower demand [22-25]. The broader mental health burden associated with sleep disturbance also intersects with prescribing pressures in the community [26]. From a systems perspective, policy incentives can shape utilization. Japan’s Diagnosis Procedure Combination (DPC) framework and contemporaneous medical fee revisions that promote generics may contribute to regional patterns, alongside environmental and demographic determinants [27,28]. Taken together, these influences suggest that circadian‐related hypnotics are not prescribed uniformly, but reflect a multifactorial interplay among environment, population aging, access, and policy.

Residual-based and standardized utilization approaches have been widely applied in health services research to identify regions where healthcare use or spending deviates from expected levels, given demographic and need-adjusted characteristics. Several studies have demonstrated that regional variation in healthcare utilization persisted after adjustment for population characteristics, indicating that residual differences may reflect inefficiency or misallocation of healthcare resources [29-31]. Following this concept, the residuals in our model represent deviations from expected prescribing patterns under comparable demographic and environmental conditions, serving as hypothesis-generating signals rather than definitive measures of inappropriate use. Practically, this residual-based indicator provides an early-warning signal for regions where prescribing exceeds expected levels and aligns with established approaches to detecting unwarranted regional variation in drug spending and prescribing quality, thereby supporting targeted stewardship, audits, and resource allocation [31,32].

Drug‐specific dynamics also matter. Lemborexant - a newer dual orexin receptor antagonist - was used more often among younger adults and demonstrates fewer falls and less residual sedation than suvorexant in clinical studies, which may drive different adoption curves across populations [33-37]. These pharmacological and demographic contrasts help explain the weaker ecological coupling to sunlight for this agent. This study also has notable strengths. It leveraged nationwide, publicly available datasets covering all 47 prefectures, ensuring comprehensive representativeness and transparency. By integrating environmental, demographic, and healthcare accessibility factors within a unified framework, the study provides a multidimensional perspective on regional prescribing behavior. The use of standardized, population-based measures, residual-based mapping, and reproducible analytical tools (R, EZR, and QGIS) enhances both robustness and replicability. These methodological strengths increase confidence that the observed associations reflect genuine macro-level prescribing tendencies, rather than sampling or analytic artifacts.

Limitations

This study has limitations. Because this was an ecological analysis, prefectural-level associations do not necessarily reflect individual-level prescribing behaviors; thus, the results should be interpreted as population-level trends, rather than causal relationships.

As an ecological analysis, it used aggregated prefectural data and cannot adjust for individual‐level confounding; future work should incorporate time‐series methods (e.g., distributed lag non‐linear models) to probe temporality and dose-response more rigorously [37,38]. While we analyzed public, nonidentifiable data according to international guidance for health‐related research, cautious interpretation is warranted when translating ecological signals into policy [39].

In conclusion, environmental and demographic determinants account for a substantial share of inter‐prefectural variance in prescriptions of melatonin and orexin receptor modulators. A residual‐based indicator offers a pragmatic tool to detect potential regional overuse and to guide climate‐sensitive, regionally optimized strategies for safer hypnotic prescribing and public health surveillance. Collectively, these results underscore the analytical and policy relevance of residual-based mapping in pharmacoepidemiology. Overall, these findings highlight the potential utility of residual-based indicators in population-level pharmacoepidemiological monitoring. From a policy perspective, the residual-based indicator could be incorporated into Japan’s existing healthcare quality- and cost-monitoring frameworks. For example, within the DPC reimbursement system, regional residual metrics could serve as early-warning signals for disproportionate prescribing or underuse, complementing ongoing audits of pharmaceutical expenditures.

Furthermore, integration with the NDB analytics platform would allow periodic updates and longitudinal monitoring of prescribing variation in response to demographic and environmental changes. Such integration would facilitate data-driven policymaking, enabling regional health authorities to design targeted interventions for rational hypnotic use, while maintaining equity and efficiency in resource allocation. This regionally adaptive surveillance aligns with existing frameworks for monitoring healthcare quality and unwarranted variation [31,32], while also supporting policy‐responsive prescribing systems under Japan’s DPC‐based reimbursement structure [27,28].

## Conclusions

This nationwide ecological analysis identified sunlight duration and population aging as key environmental and demographic determinants of regional variation in prescriptions of circadian rhythm-modulating hypnotics in Japan. These findings highlight that hypnotic prescribing patterns are not uniform, but reflect a multifactorial interplay among photic environment, age structure, healthcare access, and policy context. Residual-based indicators derived from ecological models may serve as practical tools for identifying regions of potential overuse and guiding regionally optimized, climate-sensitive strategies for safer hypnotic prescribing.

## Figures and Tables

**Figure 8:**
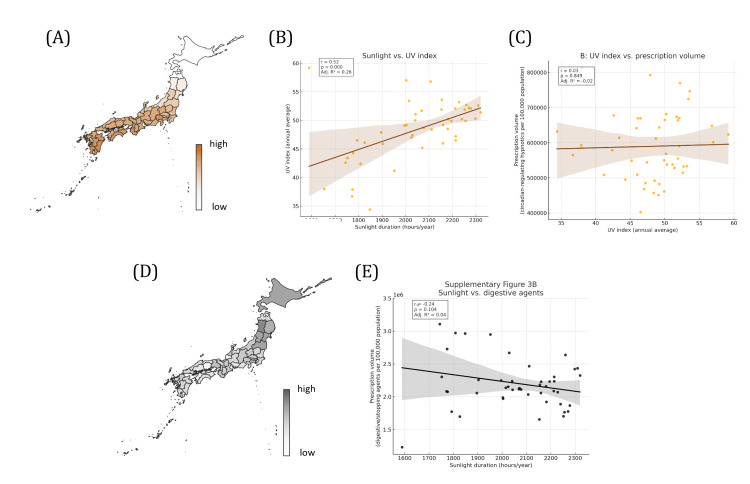
Geographic distribution and clinical correlates of ultraviolet exposure in Japan (A) Average annual ultraviolet (UV) index across Japan’s prefectures. Data were obtained from the Ministry of the Environment and the Japan Meteorological Agency (JMA). The color scale indicates relative UV intensity, with higher levels in southwestern Japan and lower levels in Hokkaido and Tohoku. (B) Association between the UV index and sunshine duration. Both datasets were from the JMA. Linear regression analysis provided the correlation coefficient (r), p-value, and adjusted R². A significant positive correlation was observed, indicating that areas with longer sunshine hours exhibit higher UV exposure. (C) Correlation between the UV index and population-adjusted prescription volumes of ramelteon, suvorexant, and lemborexant. Linear regression was conducted for each drug, showing no significant relationships, suggesting that UV exposure has a limited direct influence on prescribing behavior. (D) Population-adjusted prescription volume of intestinal regulators (e.g., biofermin), used as negative controls. Prescription data were from the National Database of Health Insurance Claims and Specific Health Checkups (NDB) and expressed per 100,000 individuals. Regional variation was visualized by color intensity, with no notable association with UV index or sunshine duration. (E) Association between intestinal regulator prescriptions and the three circadian rhythm-regulating hypnotics. Linear regression revealed no significant correlations, confirming the validity of intestinal regulators as negative controls in this ecological analysis.

**Figure 9:**
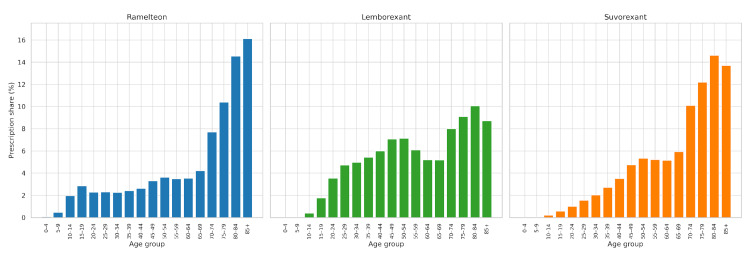
Prescription volume by age group This figure illustrates the distribution of the annual prescription volumes of ramelteon, suvorexant, and lemborexant by age group. Prescription data were obtained from the National Database of Health Insurance Claims and Specific Health Checkups of Japan (NDB) and aggregated accordingly. The prescription volume for all drugs was higher among older adults, particularly those aged ≥65 years. Lemborexant showed a distinct age distribution pattern compared with the other two agents.

**Figure 10:**
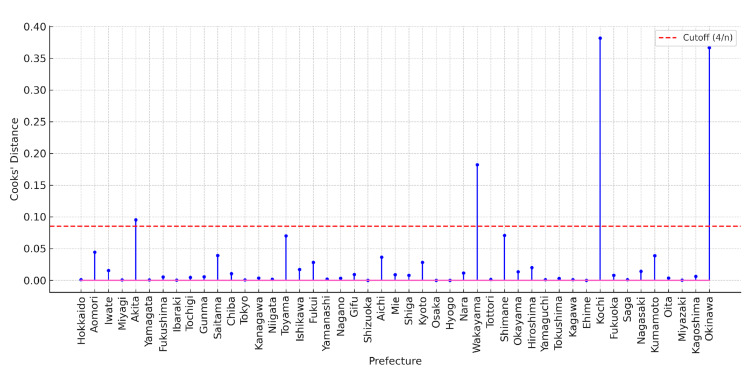
Influence diagnostics based on Cook’s distance The distribution of Cook’s distance values across all prefectures is used to assess the influence of each data point on the multivariable regression models. Prefectures with high Cook’s distance values were considered potential outliers. This analysis supported the robustness of the model and identified areas at risk of overfitting.

## Appendices

**Table 2:** Descriptive statistics for demographic and environmental variables across 47 prefectures of Japan This table summarizes prefectural-level descriptive data used for population standardization and correlation analyses. Variables include total population, aging rate (% population aged ≥65 years), annual sunlight duration, ultraviolet (UV) index, and outpatient clinic density (per 100,000 population). All data were obtained from official national sources: the Statistics Bureau of Japan (Population Estimates 2022, Survey of Medical Institutions 2022), the Japan Meteorological Agency (Climate Statistics 2022), and the Ministry of the Environment (UV Environmental Health Manual 2022).

Variable	Mean ± SD	Min	Max	Source
Annual sunlight duration (hours)	2,047.1 ± 194.9	1,589.00	2,320.00	Japan Meteorological Agency, Climate Statistics 2022 [15]
Mean UV index	4.84 ± 0.52	3.44	5.92	Japan Meteorological Agency & Ministry of the Environment, UV Environmental Health Manual 2022 [16]
Population aged ≥65 years (%)	31.35 ± 3.27	22.81	38.64	Statistics Bureau of Japan, Population Estimates 2022 [17]
Total population (persons)	2,658,426 ± 2,793,536	544,000	14,038,000	Statistics Bureau of Japan, Population Estimates 2022 [17]
Number of outpatient clinics (per 100,000 population)	84.8 ± 12.1	61.3	114.1	Statistics Bureau of Japan, Survey of Medical Institutions 2022 [40]

**Table 3:** Overview of data preprocessing and diagnostic procedures used in this study This table outlines the analytical workflow, including variable standardization, outlier detection, multicollinearity assessment (VIF < 5), Cook’s distance threshold (4/n), and diagnostic visualization (residual and Q-Q plots). Outlier influence was assessed using Cook’s distance (threshold: 4/n). Residual and Q-Q plots were visually inspected to ensure linearity and homoscedasticity. All analyses were performed using R (version 4.3.0) and EZR (version 1.61).

Procedure	Description	Diagnostic threshold/software	Purpose
Population standardization	Prescription volumes for ramelteon, suvorexant, and lemborexant were standardized per 100,000 population using official prefectural census data (2022).	Statistics Bureau of Japan (2022)	Enables inter-prefectural comparison of prescription volumes
Variable transformation	All variables (sunlight duration, aging rate, and clinic density) were analyzed as continuous values without log transformation to maintain interpretability.	R 4.3.0/EZR 1.61	Preserves linear scale for interpretation
Multicollinearity check	Variance inflation factor (VIF) was computed for each explanatory variable.	VIF < 5 for all variables	Confirms absence of multicollinearity
Outlier influence	Cook’s distance was calculated for each prefecture in multivariable models. Values > 4/n were flagged as influential and checked in sensitivity analysis.	Cook’s D > 4/n	Detects high-influence prefectures and ensures robustness
Model diagnostics	Residual-versus-fitted and Q–Q plots were inspected visually.	R base graphics tools	Verifies linearity and homoscedasticity of models

**Table 4:** Univariate regression analysis of prescription volume and explanatory variables This table presents the relationships between prefectural-level prescription volumes of ramelteon, suvorexant, and lemborexant and three explanatory variables: annual sunlight duration, aging rate (percentage of the population aged ≥65 years), and number of outpatient clinics. A significant negative association was observed between ramelteon use and duration of sunshine.

Dependent variable	Factor	Standardized β	Pearson r	p-value	95% CI
Lemborexant	UV index	1284.83	0.1562	0.294301	-1153.91 to 3723.57
Lemborexant	Clinic density	0.749	0.213	0.150498	-0.28 to 1.78
Lemborexant	Sunlight duration	-30.442	-0.139	0.351305	-95.54 to 34.66
Lemborexant	Aging rate	310504.08	0.2381	0.107004	-69743.96 to 690752.12
Suvorexant	UV index	484.781	0.0575	0.701075	-2042.60 to 3012.16
Suvorexant	Clinic density	1.22	0.3385	0.01996	0.20 to 2.24
Suvorexant	Sunlight duration	-42.496	-0.1893	0.202522	-108.68 to 23.69
Suvorexant	Aging rate	860111.964	0.6433	1.08 x 10^-6^	552786.63 to 1167437.30
Ramelteon	UV index	-1243.555	-0.2449	0.097089	-2721.70 to 234.59
Ramelteon	Clinic density	-0.152	-0.07	0.639895	-0.80 to 0.50
Ramelteon	Sunlight duration	-68.002	-0.503	0.000314	-103.08 to -32.92
Ramelteon	Aging rate	389284.07	0.4835	0.000576	177672.68 to 600895.46
Digestive Agent	UV index	-49165.805	-0.6225	2.98 x 10^-6^	-67724.29 to -30607.32
Digestive Agent	Clinic density	2.562	0.0759	0.612122	-7.54 to 12.67
Digestive Agent	Sunlight duration	-504.663	-0.24	0.104193	-1117.57 to 108.24
Digestive Agent	Aging rate	2444803.269	0.1952	0.188515	-1242998.77 to 6132605.31

**Table 5:** Multivariable regression output for each drug This table summarizes the multivariate regression results corresponding to Figure 4, where annual sunlight duration, aging rate, and outpatient clinic density were included as explanatory variables. Standardized regression coefficients (β), 95% confidence intervals (CIs), p-values, and the adjusted R² of the model are reported for each drug.

Drug	Factor	Beta	95% CI	p-value	Adjusted R^2^
Ramelteon	Sunlight	-10855.939	-17615.508 to -4096.370	0.0023	0.37
Ramelteon	Aging Rate	10765.491	4188.358 to 17342.624	0.0019	0.37
Ramelteon	Clinic Density	-798.645	-7616.687 to 6019.397	0.814	0.37
Suvorexant	Sunlight	-7477.124	-18047.687 to 3093.438	0.161	0.44
Suvorexant	Aging Rate	23824.58	13539.308 to 34109.851	0.0000	0.44
Suvorexant	Clinic Density	11650.585	988.582 to 22312.587	0.033	0.44
Lemborexant	Sunlight	-7916.033	-21356.747 to 5524.681	0.241	0.05
Lemborexant	Aging Rate	6299.674	-6778.285 to 19377.633	0.337	0.05
Lemborexant	Clinic Density	10094.076	-3462.905 to 23651.057	0.141	0.05
Digestive Agent	Sunlight	-105636.994	-235965.905 to 24691.916	0.109	0.03
Digestive Agent	Aging Rate	47362.27	-79449.165 to 174173.705	0.455	0.03
Digestive Agent	Clinic Density	53744.268	-77712.042 to 185200.577	0.414	0.03
